# The Nuclear Localization Signal of Porcine Circovirus Type 4 Affects the Subcellular Localization of the Virus Capsid and the Production of Virus-like Particles

**DOI:** 10.3390/ijms25052459

**Published:** 2024-02-20

**Authors:** Jiawei Zheng, Nan Li, Xue Li, Yaqi Han, Xinru Lv, Huimin Zhang, Linzhu Ren

**Affiliations:** 1 College of Animal Sciences, Key Lab for Zoonoses Research, Ministry of Education, Jilin University, 5333 Xi’an Road, Changchun 130062, China; zhengjw21@mails.jlu.edu.cn (J.Z.);; 2 Changchun Veterinary Research Institute, Chinese Academy of Agricultural Sciences, 666 Liuying West Road, Changchun 130122, China; linan226@126.com; 3 College of Veterinary Medicine, Yunnan Agricultural University, Kunming 650201, China

**Keywords:** porcine circovirus 4 (PCV4), nuclear localization signal (NLS), capsid (Cap)

## Abstract

Porcine circovirus 4 (PCV4) is a newly identified virus belonging to PCV of the *Circoviridae* family, the *Circovirus* genus. We previously found that PCV4 is pathogenic in vitro, while the virus’s replication in cells is still unknown. In this study, we evaluated the N-terminal of the PCV4 capsid (Cap) and identified an NLS at amino acid residues 4–37 of the N-terminus of the PCV4 Cap, ^4^RSRYSRRRRNRRNQRRRGLWPRASRRRYRWRRKN^37^. The NLS was further divided into two fragments (NLS-A and NLS-B) based on the predicted structure, including two α-helixes, which were located at ^4^RSRYSRRRRNRRNQRR^19^ and ^24^PRASRRRYRWRRK^36^, respectively. Further studies showed that the NLS, especially the first α-helixes formed by the NLS-A fragment, determined the nuclear localization of the Cap protein, and the amino acid ^4^RSRY^7^ in the NLS of the PCV4 Cap was the critical motif affecting the VLP packaging. These results will provide a theoretical basis for elucidating the infection mechanism of PCV4 and developing subunit vaccines based on VLPs.

## 1. Introduction

Porcine circoviruses (PCVs) belong to the *Circoviridae* family of the *Circovirus* genus. They are characterized by non-enveloped, icosahedral virions composed of circular single-stranded DNA genomes with a diameter of approximately 17–20 nm. Currently, four types of PCVs have been identified: PCV1, PCV2, PCV3, and PCV4 [1,2]. PCV1 is non-pathogenic, while PCV2 is the primary pathogen associated with porcine circovirus-associated diseases (PCVADs) [3,4]. PCV3 was first discovered in 2016 in America, and PCV4 is a novel type of PCV first reported in Hunan Province, China, in 2019, associated with clinical symptoms such as respiratory distress, porcine dermatitis, and nephropathy syndrome (PDNS) [5,6]. Since then, both PCV3 and PCV4 have been detected in other pig-rearing provinces in China and other countries, indicating their potential wide distribution among pig farms [7,8].

The N-terminal amino acids of the PCV2 Cap protein constitute a nuclear localization signal (NLS) [9]. Existing research shows that the NLS of PCV2 is involved in viral replication and translation [10,11,12]. The PCV4 genome mainly encodes two open reading frames, one encoding the replication enzyme (rep) and the other encoding the capsid protein (Cap) [5]. We previously found that PCV4 shares a higher nucleotide identity (66.9%) with mink circovirus but a lower homology (43.2–51.5%) with other PCVs [2]. Although the PCV4-Cap sequence differs significantly from other PCVs, the NLS sequence contains conserved amino acids and shows high similarity, responsible for transporting cellular proteins into the cell nucleus [2,13,14]. Therefore, the study of the PCV4 NLS is vital for understanding its role in the PCV4 life cycle and for PCV family research. It is worth noting that purified Cap can self-assemble into virus-like particles (VLPs) in vitro [15]. However, the specific role of the N-terminal segment of the PCV4 Cap, including the NLS, in the PCV4-VLP assembly remains unclear.

In this study, we aimed to explore the structural role of the N-terminal segment of the PCV4 Cap in the PCV4 assembly and its involvement in mediating the nuclear entry process of PCV4. We constructed various N-terminal-truncated forms of Cap and compared their VLP packaging efficiencies. Our findings demonstrate that the N-terminal ^4^RSRY^7^ is critical in Cap’s self-assembly into VLPs. Furthermore, the N-terminal ^4^RSRYSRRRRNRRNQRR^19^ is crucial for mediating the nuclear entry of the PCV Cap, potentially impacting PCV4 replication.

## 2. Results

### 2.1. Subcellular Localization of PCV4 Cap and Identification of the NLS

**Figure 1:**
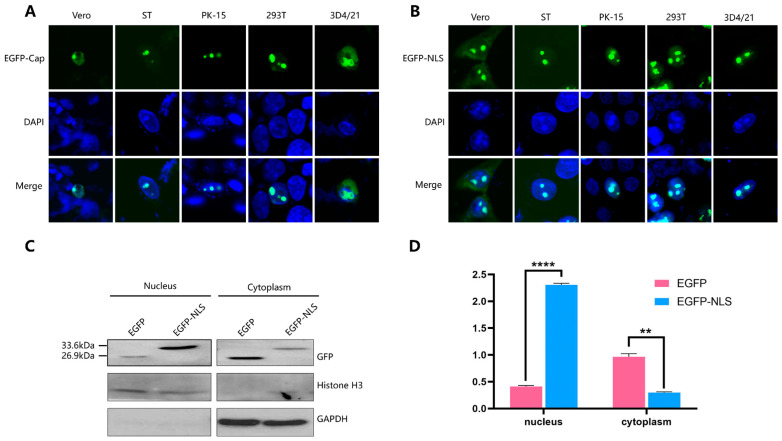
Subcellular localization of PCV4 Cap and identification of the NLS. (**A**) Subcellular localization of PCV4 Cap. The recombinant plasmid pEGFP-Cap was transfected into Vero, ST, PK-15, HEK-293T, and 3D4/21 cells, followed by a laser confocal microscope examination (63×). (**B**) Subcellular localization of NLS-fused EGFP. The plasmid pEGFP-NLS was transfected into Vero, ST, PK-15, HEK-293T, and 3D4/21 cells, followed by laser confocal microscope evaluation (63×). (**C**,**D**) Distribution of EGFP fused with or without the NLS in the nucleus and cytoplasm. PK-15 cells were transfected with pEGFP-N1 or pEGFP-NLS. Levels of GFP were evaluated by Western blot (**C**) and quantified by gray analysis with normalization to histone H3 or GAPDH using ImageJ Version 1.8.3 (**D**). Western blot was performed using GFP ANTIBODY, anti-His-tag antibody, or histone H3 antibody as the primary antibody and HRP-conjugated goat anti-mouse IgG(H+L) as the secondary antibody. GAPDH was used as an additional loading control, and histone H3 was an indicator of nuclear proteins. Unprocessed original scans of the gel can be found in Supplementary Figure S1. **, *p* < 0.01; ****, *p* < 0.0001.

To confirm the intracellular distribution of the PCV4 Cap, the full-length sequence of the Cap protein was cloned into the green fluorescent protein (EGFP)-expressing plasmid pEGFP-N1, followed by transfecting the recombinant plasmid into Vero, ST, PK-15, HEK-293T, and 3D4/21 cells. The results demonstrated that the green fluorescence (EGFP-Cap) gathered within the cell nuclei of Vero, ST, PK-15, HEK-293T, and 3D4/21 cells (Figure 1A). These results indicate that an NLS is also located in the PCV4 Cap protein, which may play similar roles to that of PCV2.

To identify the NLS of the PCV4 Cap, the Cap protein sequence was analyzed using the online programs cNLS mapper and NovoPro. An NLS was predicted at amino acid residues 4–37 of the N-terminus of the PCV4 Cap, ^4^RSRYSRRRRNRRNQRRRGLWPRAS RRRYRWRRKN^37^. Furthermore, the NLS contained two α-helixes at ^4^RSRYSRRRRNRRNQRR^19^ and ^24^PRASRRRYRWRRK^36^, respectively.

Moreover, the predicted NLS of the PCV4 Cap was linked to the *egfsp* gene of the pEGFP-N1 plasmid to obtain the recombinant eukaryotic expression plasmid pEGFP-NLS, followed by transfecting into Vero, ST, PK-15, HEK-293T, and 3D4/21 cells. As shown in Figure 1B, almost all of the green fluorescence was detected in the nucleus of the pEGFP-NLS-transfected group (EGFP-NLS), which is similar to the results of the fused protein EGFP-Cap in Figure 1A. Furthermore, the EGFP fused with the NLS was mainly distributed in the nucleus. At the same time, the EGFP without the NLS was primarily located in the cytoplasm (Figure 1C,D). These results further confirm that the PCV4 Cap contains an NLS, which may be critical for the Cap protein to enter the nucleus.

### 2.2. Truncated NLS Affects the Subcellular Localization of PCV4 Cap Protein

**Figure 2:**
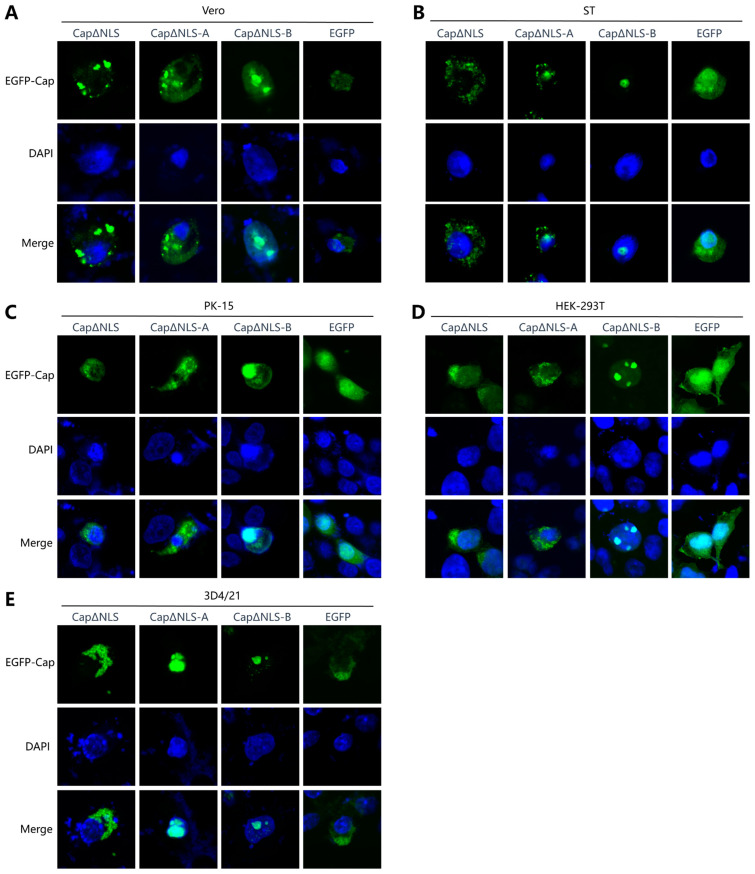
Expressions of different truncated Cap proteins in cells. Cells were transfected with plasmids pEGFP-N1, pEGFP-Cap∆NLS, pEGFP-Cap∆NLS-A, and pEGFP-Cap∆NLS-B, respectively, followed by laser confocal microscope evaluation (63×). (**A**) Vero cell. (**B**) ST cell. (**C**) PK-15 cell. (**D**) HEK-293T cell. (**E**) 3D4/21 cell.

Since the NLS contains two α-helixes, the influence of the two α-helixes on the nucleus entry of the NLS was evaluated. The results show that the Cap protein could not enter the nucleus after the NLS (CapΔNLS) was removed entirely (Figure 2), which was contrary to the result when the NLS was preserved (Figure 1) and further confirms that the NLS is necessary for the Cap protein to enter the nucleus. 

Furthermore, the green fluorescence was mainly located in the nuclei of the cells transfected with CapΔNLS-B, which expressed EGFP-fused Cap with the second α-helixes of the NLS deleted. These results suggest that the NLS-B fragment may not be essential for the nuclear import of the Cap protein. On the contrary, truncated Caps lacking NLS-A (the CapΔNLS-A group) were only distributed around the nuclei of Vero, ST, 293T, and PK-15 cells, indicating that the NLS-A fragment plays a more critical role in mediating the protein into the nucleus than NLS-B. The above results demonstrate that the NLS, especially the first α-helixes formed by the NLS-A fragment, determines the nuclear localization of the Cap protein.

### 2.3. Truncated NLS Affects the Production of Virus-like Particles

**Figure 3:**
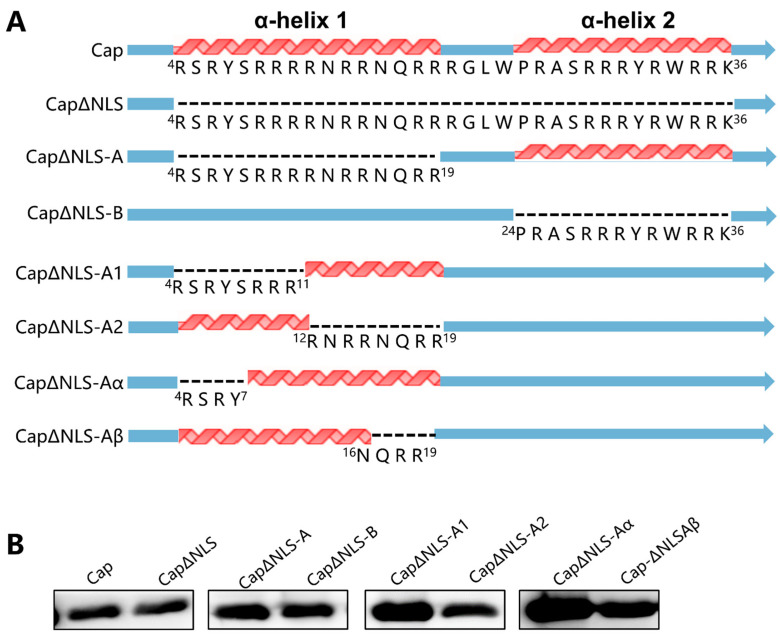
Truncated NLS did not affect the expression of Cap. (**A**) Schematic diagram of the PCV4 Cap with or without truncated NLS. The dashed line indicates deleted amino acids. (**B**) Expression of truncated forms of PCV4 Cap proteins. Western blot was performed using an anti-His-tag antibody as the primary antibody and HRP-conjugated goat anti-mouse IgG(H+L) as the secondary antibody. Unprocessed original scans of the gel can be found in Supplementary Figure S2.

Since the Cap protein is the only structural protein of PCV, which can form an icosahedral capsid composed of 60 subunits of the PCV Cap [16,17], the influence of the PCV4 NLS and its truncated forms on the formation of virus-like particles was evaluated. As shown in Figure 3A, eight constructs of the PCV4 capsid with different truncated forms were constructed, followed by an expression evaluation of the protein. All the constructs can be effectively expressed in *E. coil* (Figure 3B). 

**Figure 4:**
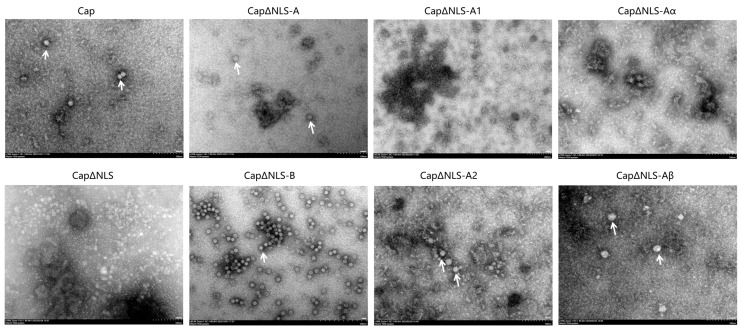
VLPs prepared from soluble recombinant proteins. VLPs were stained with 1% phosphotungstic acid and examined using a Hitachi TEM system HC-1. Scale bar = 200 nm. Arrow indicates the target VLP.

However, the packaging rate of VLPs was diverse among the constructs. As shown in Figure 4, the PCV4 capsid with full-length amino acids (the Cap group) and the Cap with NLS-B deletion (CapΔNLS-B) could be efficiently assembled into VLPs. At the same time, the Cap with NLS-deletion (CapΔNLS) could not typically be packaged into mature VLPs. Furthermore, immature VLPs were observed in the CapΔNLS-A group, which contained an NLS-A deletion in the NLS, indicating that the NLS-A, but not NLS-B, may also affect the formation of VLPs. Therefore, the NLS-A was further divided into two fragments, NLS-A1 and NLS-A2. As expected, the PCV4 Cap with NLS-A1 deleted (CapΔNLS-A1) could not form VLPs, while stable VLPs were detected in the group with NLS-A2 deleted (CapΔNLS-A2), indicating that NLS-A1 contains the crucial sequence of VLP packaging. Subsequently, no mature VLPs were detected in the truncated CsapΔNLS-Aα, which had deletions at ^4^RSRY^7^ of the first α-helix of the NLS (NLS-A). In contrast, stable VLPs were observed in the control group CapΔNLS-Aβ, which contained four amino acid deletions at the C-terminal of NLS-A (^16^NQRR^19^). These results indicate that the amino acid ^4^RSRY^7^ in the NLS of the PCV4 Cap is the critical motif affecting VLP packaging.

## 3. Discussion

Recently, PCV4 has been widely spreading in China and other countries, and the infection rate has been increasing year by year [18,19,20,21]. Currently, no effective treatment or commercial vaccine is available in the domestic and foreign markets. There are few studies on the PCV4 Cap protein, but it is well-established that the Cap protein plays a vital role in virus replication and assembly in the PCV family [22,23]. As the only structural protein of PCV4, the PCV4 Cap is highly conserved. Therefore, the study of the structure and function of the PCV4 Cap is of great significance for the subsequent research of PCV4. This study aimed to investigate the effect of the NLS of the PCV4 Cap protein on Cap protein expression, VLP packaging, and the intracellular localization of the Cap protein.

NLSs are divided into classical NLSs and non-classical NLSs [24]. The difference between the two is that non-classical NLSs do not have the sequence characteristics of classical NLSs or contain negatively charged amino acid residues. Classical NLSs are also divided into monopartite-type NLSs and bipartite-type NLSs. The monopartite-type NLSs contain only one cluster of positively charged amino acid residues. In contrast, the bipartite-type NLSs comprise two clusters of positively charged amino acid residues separated by a small spacer sequence. Similar to the structure of the Cap proteins of PCV2 and PVC3 [12], we also found in this study that an NLS is located at the N-terminal end of the PCV4 Cap protein, consisting of 34 amino acids. The PCV4 NLS can be divided into two parts, NLS-A and NLS-B, due to its two α-helices connected by intermediate transition flex residues. Therefore, it is speculated that the NLS of the PCV4 Cap protein is a classical nuclear localization signal with numerous two-component nuclear localization signals.

Subsequently, we assessed the intracellular localization of fusion proteins expressing EGFP and a truncated PCV4 Cap. As expected, when the NLS segment was wholly deleted, or the NLS-A segment was deleted, the intracellular localization of the fusion protein became cytoplasmic, significantly different from that of the wild-type EGFP-Cap fusion proteins localized in the nucleus. Furthermore, when the NLS region of the PCV4 Cap protein was fused to the N-terminus of EGFP alone, the recombinant protein could be transferred into the nucleus. Thus, the subcellular localization of PCV4 CapΔNLS and the fact that recombinant EGFP-NLS can enter the nucleus confirm the decisive role of the NLS in the subcellular localization of the PCV4 Cap protein. Moreover, the results demonstrate that the green fluorescence gathered in dots within the nuclei of HEK-293T, PK-15, and ST cells (Figure 1A). However, the green fluorescence in the nuclei of 3D4/21 and Vero cells was scattered. This result indicates that the PCV4 Cap protein can be further localized in the nuclei of PK-15, HEK-293T, and ST cells, suggesting that nucleolar localization signals (NoLSs) also exist in the Cap protein. As reported, NLSs usually overlap with NoLSs [25,26,27]. This nucleolus localization may be related to processes such as the replication and packaging of PCV4 virion. Therefore, the analysis of the NoLS region and its contribution will be further investigated.

Intriguingly, while the above findings were validated across various cell lines, they did not yield analogous results in the 3D4/21 cell line. In 3D4/21 cells, the truncated Cap proteins devoid of NLS-A persistently exhibited nuclear localization, suggesting that there are other nuclear import mechanisms besides the NLS in 3D4/21 cells, which needs further investigation (Figure 2). For example, the β-catenin protein has three different transport sequences: the N-terminal tail, the C-terminal tail, and the armadillo repeats [28], which have no apparent sequence homology, but the hydrophobic regions in the N- and C-terminal regions are auxiliary to the nuclear entry [29]. Similarly, Lyst et al. found that the nuclear protein MeCP2 (methyl-CpG-binding protein 2) may pass through the nuclear pore complex in an NLS-independent manner and import proteins, which contain two sequence-specific DNA-binding motifs, AT-hook1 and the methyl-CpG-binding domain (MBD) [30]. 

Beyond its pivotal role in the intracellular localization of PCV, prior research has also elucidated the impact of NLSs on the assembly of PCV VLPs. For example, Mo et al. investigated the effect of the NLS-containing region at the N-terminus of the PCV2 Cap protein on the formation of PCV2 VLPs [22]. It was demonstrated that NLS truncation could only form unstable and easily degradable PCV2 VLPs. The instability of VLPs formed by NLS truncation was due to the lack of an α-helix in the NLS interacting with NLS-B on the neighboring Cap proteins. In this study, various NLS-truncated Cap proteins with His tags were expressed by the *E. coli* expression system to evaluate its effect on assembling PCV4 VLPs. The results show that the residues ^4^RSRY^7^ of the NLS were the critical amino acid residues affecting the assembly of VLPs. This result demonstrates that the NLS fragment at the N-terminus of the PCV4 Cap plays a crucial role in forming VLPs.

*E. coli* is a well-established system for expressing recombinant proteins [31], offering advantages such as ease of use, rapid growth, and cost-effectiveness. These advantages make it a practical choice for producing large quantities of proteins, like the PCV capsid protein, for experimental purposes [32]. Furthermore, the *E. coli* expression system is a classic choice for expressing viral proteins and VLPs [33]. Its widespread use for expressing VLPs in PCV studies is well-documented [32]. The *E. coli* system is particularly advantageous for these purposes due to its ability to facilitate high-level expression, and the simplicity of purifying expressed proteins, making it an ideal choice for producing PCV4 capsid proteins and studying the effects of NLS truncation on VLP formation and Cap protein detection. Because the NLS at the N-terminus of Cap is rich in arginine residues, the expression of the Cap protein is often enhanced by truncating the NLS fragment in the *E. coli* system [34,35]. However, our study found that the truncation of NLS fragments affects the structural stability of VLPs. VLPs, on the other hand, do not have viral genetic material, and cannot replicate and infect the host, but they can form a spatial three-dimensional structure similar to that of natural viral particles, which can effectively induce immune protection in the organism. Meanwhile, VLPs are more suitable for the development of serological diagnostic tests due to their higher safety and ease of use. VLP-based enzyme-linked immunosorbent assays (ELISAs) are widely used to measure antibodies or neutralizing epitopes. This may result in the use of recombinant proteins with truncated NLSs to prepare antibodies or vaccines. However, due to the structural instability of VLPs, they may not be as effective as wild strains, which may affect vaccine resistance or the binding of antibodies to virions.

Moreover, the nuclear translocation of viral proteins mediated by NLS sequences is essential in viral replication, packaging processes, and immune escape. For example, in Bombyx mori nucleopolyhedrovirus (BmNPV), the polyhedron (POLH) NLS was found to have a significant effect on the number, intracellular localization, and morphology of occlusion bodies (OBs) [36]. Recombinant NDV with mutations in the NLS of the M protein is reduced in pathogenicity, and the NLS of the M protein in NDV promotes NDV replication by increasing the efficiency of viral RNA synthesis and transcription as well as by inhibiting host cell transcription [37,38]. Mutations in the NLS in the core protein of the Japanese encephalitis virus (JEV) reduce both viral replication in mammalian cells in vitro and the pathogenesis of JEV-induced encephalitis in vivo [39]. Similarly, nuclear-localized influenza A virus (IAV) nucleoprotein N-terminal deletion mutants lack viral mRNA translation and exhibit defects in forming functional viral ribonucleoproteins, leading to delayed replication in IAV-infected cells [40]. In addition, recent studies have shown that nuclear import of the rabies virus P protein facilitates the inhibition of host gene transcription, regulates viral genome replication and transcription, and disrupts antiviral signaling pathways [41,42]. This result shows that NLS sequences are necessary in the virus infection process. However, the function played by the NLS of the capsid during PCV4 infection needs further study.

## 4. Materials and Methods

### 4.1. Construction of the Eukaryotic Expression Vectors

The full-length sequence of the PCV4 capsid gene or its predicted NLS sequence (GenBank No.: MT311854.1) was synthesized and cloned into the eukaryotic expression plasmid pEGFP-N1 (Biosciences Clontech, Mountain View, CA, USA) using *Xho* I and *Hin*d III, resulting in the eukaryotic expression plasmid pEGFP-Cap or pEGFP-NLS. Then, recombinant eukaryotic expression plasmids with truncated NLS sequences were constructed using primers (Supplementary Table S1) by site-directed mutagenesis techniques (Q5^®^ Site-DirectedMutagenesis Kit, NEB, Lpswich, MA, USA) and named pEGFP-Cap∆NLS, pEGFP-Cap∆NLS-A, and pEGFP-Cap∆NLS-B, respectively. pEGFP-Cap∆NLS express a fused EGFP-Cap without the NLS, while the plasmids pEGFP-Cap∆NLS-A and pEGFP-Cap∆NLS-B express a fused EGFP-Cap with a truncated Cap lacking NLS-A or NLS-B, respectively. 

Site-directed mutagenesis techniques were conducted by PCR amplification using pEGFP-Cap as a template. PCR amplification was performed in a final volume of 50 μL, containing 25 μL of 2× MasterMix (ABM, Shanghai, China), 1 μL of each forward and reverse primer (10 μM of each primer; Jilin Kumei Biotechnology Co., Ltd., Jilin, China), 2 μL of the DNA template (20 ng/μL), and 21 μL of ddH_2_O. The PCR program consisted of an initial denaturation step at 94 °C for 5 min, followed by 35 cycles of denaturation at 94 °C for 30 s, annealing at 53 °C for 30 s, and extension at 72 °C for 45 s. A final extension step at 72 °C for 10 min was performed, followed by a final hold at 4 °C. The PCR products were linked into the expression vector pEGFP-N1 via *Xho* I and *Kpn* I, respectively.

### 4.2. Laser Confocal Microscope

Human embryonic kidney (HEK-293T) cells, African green monkey kidney epithelial (Vero) cells, porcine kidney (PK-15) cells, porcine testis (ST) cells, and porcine alveolar macrophage (3D4/21) cells were seeded in 6-well dishes and incubated at 37 °C in a 5% CO_2_ cell culture incubator to 70–90% confluency. Cells were transfected with the eukaryotic expression plasmids pEGFP-N1, pEGFP-Cap, pEGFP-NLS, pEGFP-Cap∆NLS, pEGFP-Cap∆NLS-A, and pEGFP-Cap∆NLS-B, respectively, using a lipid-based transfection method [43] (Hieff Trans^®^ Liposomal Transfection Reagent, Yeasen, Shanghai, China) according to the manufacturer’s instructions. 

Briefly, 10 μL of the lipid-based transfection reagent was added to 250 μL of Opti-MEMTM medium and incubated at room temperature for 5 min. An amount of 2–4 μg of plasmid DNA was added to 250 μL of Opti-MEMTM medium and mixed. Then, the reagents obtained above were mixed and incubated at room temperature for 20 min and added to a plate at 37 °C for 48 h. The cell culture supernatant was discarded, and the cells were fixed by adding 80% acetone and incubating at −80 °C for 2 h. After three washes with PBST, the cells were treated with DAPI solution (Servicebio, Wuhan, China) for 5 min in a dark room. Then, the cells were washed three times with PBST and examined using a laser confocal microscope (Zeiss LSM880 with Airyscan, Zeiss, Jena, Germany).

### 4.3. NLS Prediction

The PCV4 capsid sequence was analyzed to identify the NLS using the online programs cNLS mapper (http://nls-mapper.iab.keio.ac.jp/cgi-bin/NLS_Mapper_form.cgi (accessed on 20 September 2022) and NovoPro (https://www.novopro.cn/tools/secondary-structure-prediction.html (accessed on 20 September 2022) according to the protocols described on the websites.

### 4.4. SDS-PAGE and Western Blotting

The total protein was separated by SDS-PAGE with 10% stacking gel and 12% separating gel at 80 V for 2 h and then transferred onto a PVDF membrane at 90 V for 55 min at 4 °C. Then, the membrane was blocked with 5% skimmed milk (diluted in TBS-T) at room temperature in a shaker for 1 h. After 3 washes with TBS-T, the membrane was incubated with the mouse GFP antibody [44] (1:4000, Proteintech Group, Wuhan, China), anti-GAPDH rabbit monoclonal antibody (1:30000, Boster, Wuhan, China) [45], or histone H3 antibody [46] (1:1000, Wanleibio, Shenyang, China) at room temperature for 2 h, followed by 3 washes with TBS-T. After that, the membrane was incubated with HRP-conjugated goat anti-mouse IgG(H+L) [47] (1:1000, Beyotime, Shanghai, China) or HRP-labeled goat anti-rabbit IgG(H+L) (1:1000, Beyotime, Shanghai, China) at room temperature for 1.5 h. Subsequently, the membrane was treated with ECL SuperSignal Solution [48] (Beyotime, Shanghai, China), and the protein bands were detected using a Bioanalytical Imaging System c600 (Azure Biosystems, Dublin, CA, USA).

### 4.5. Separation of Cytoplasmic and Nuclear Components

PK-15 cells were seeded in a 6-well plate overnight and transfected with pEGFP-NLS or pEGFP-N1 for 48 h, and then washed with PBS once. The cells were scraped from the plate with a cell scraper, transferred to PE tubes, and centrifuged. The cell pellet was collected, and nuclear and cytoplasmic proteins were extracted using the Nuclear and Cytoplasmic Protein Extraction Kit [49] (Beyotime, Shanghai, China) according to the manufacturer’s instructions. 

Briefly, the cell pellet was treated with 200 μL (per 20 μL of the cell pellet) of pre-cooled cell lysis buffer A containing PMSF and vortexed for 5 s to entirely suspend and disperse the cell pellet, followed by incubation on ice for 10–15 min. Then, 10 μL of pre-cooled cell lysis buffer B was added to the tube, vortexed at maximum speed for 5 s, and incubated on ice for 1 min. After that, the tube was vortexed at maximum speed for 5 s and centrifuged at 4 °C and 12,000–16,000 rpm for 5 min. The supernatant containing the extracted cytoplasmic proteins was collected and transferred immediately to a pre-chilled PE tube. 

The residual supernatant was removed carefully for the remaining samples in the centrifuge tube, and the precipitate was resuspended with 50 μL of a cell nuclear extraction buffer containing PMSF. After vortexing at maximum speed for 15–30 s, the tube was incubated on ice for 30 min and vortexed at maximum speed for 15–30 s every 1–2 min. Then, the tube was centrifuged at 4 °C and 12,000–16,000 rpm for 10 min, and the supernatant containing the extracted nuclear proteins was immediately transferred to a pre-chilled PE tube. The extracted proteins were used immediately or stored at −80 °C.

### 4.6. Construction of Prokaryotic Expression Vectors and Expression Analysis

The viral capsid genes were synthesized based on the sequences of PCV4 (GenBank no.: MT311854.1) and cloned into pET-28a (+) to generate the recombinant plasmids pET-28a-PCV4Cap. The remaining plasmids, including pET-28a-PCV4CapΔNLS, pET-28a-PCV4capΔNLS-A, pET-28a-PCV4capΔNLS-B, pET-28a-PCV4CapΔNLS-A1, pET-28a-PCV4CapΔNLS-A2, pET-28a-PCV4CapΔNLS-Aα, and pET-28a-PCV4CapΔNLSAβ, were generated with the primers described in Supplementary Table S1 using site-directed mutagenesis techniques (Q5^®^ Site-DirectedMutagenesis Kit, NEB, Lpswich, MA, USA) according to the manufacturer’s instructions. The recombinant plasmids were transformed into *E. coli* Rosetta (DE3)-competent cells and positive clones were identified via PCR and sequencing. 

The positive bacteria were cultured in an LB medium at 37 °C to obtain the recombinant protein. When the optical density at 600 nm (OD600) reached approximately 0.6–0.8, the cultures were induced with IPTG (0.7 mM) overnight at 16 °C. The induced bacterial culture was collected by centrifugation at 4 °C and 8000 rpm for 3 min to obtain the cell pellet. The pellet was washed twice with PBS (pH 8.0) and resuspended in 30 mL of pH 8.0 PBS. Subsequently, 1 mg/mL of lysozyme was added, and the suspension was incubated at 37 °C for 30 min. The resulting mixture was centrifuged to separate the supernatant from the cellular debris. Then, the pellet was resuspended in 30 mL of Binding Buffer (pH 8.0), and a final concentration of 1 mM phenylmethanesulfonyl fluoride (PMSF, Boster, Wuhan, China) was added on ice. The suspension was subjected to ultrasonication at 100 W for 3 s with a 3 s pause, for a total duration of 1.5 h, to facilitate cell disruption. Finally, the mixture was centrifuged at 4°C and 12,000 rpm for 30 min to collect the supernatant, which served as the crude protein solution.

The obtained crude protein solution was filtered through a 0.45 μm filter and purified using a Ni-NTA purification resin pre-packed column (HisPur Ni-NTA, Thermo Scientific, Waltham, MA, USA) according to the protocol described previously in [34]. The protein was collected and evaluated by SDS-PAGE and Western blotting.

### 4.7. Transmission Electron Microscope (TEM) Assay

The VLPs were stained with 1% phosphotungstic acid and examined using a Hitachi TEM system HC-1 (Hitachi, Tokyo, Japan) operated at 80 kV according to the protocol described previously in [2,34].

### 4.8. Statistical Analysis

Statistical analysis was performed using GraphPad Prism 9.5 (GraphPad Software, San Diego, CA, USA). All data were represented as means ± standard deviations. Two-way ANOVA statistical analysis was conducted using GraphPad. **, *p* < 0.01; ****, *p* < 0.0001.

## 5. Conclusions

In this study, we evaluated the N-terminal of the PCV4 capsid and identified an NLS at amino acid residues 4–37 of the N-terminus of the PCV4 Cap, 4RSRYSRRRRNRRNQRRRGL WPRASRRRYRWRRKN37. The NLS was further divided into two fragments based on the predicted structure, including two α-helixes located at 4RSRYSRRRRN RRNQRR19 and 24PRASRRRYRWRRK36, respectively. Further studies showed that the NLS, especially the first α-helixes formed by the NLS-A fragment, determines the nuclear localization of the Cap protein, and the amino acid 4RSRY7 in the NLS of the PCV4 Cap is the critical motif affecting VLP packaging.

## Data Availability

All data generated or analyzed during this study are included in this published article.

## Supplementary Materials

The following supporting information can be downloaded at https://www.mdpi.com/article/10.3390/ijms25052459/s1.
