# Supplementary material for: The Nuclear Localization Signal of Porcine Circovirus Type 4 Affects the Subcellular Localization of the Virus Capsid and the Production of Virus-like Particles
Source: Int J Mol Sci. 2024 Feb 20;25(5):2459. doi: 10.3390/ijms25052459 (PMC10930891; doi:10.3390/ijms25052459)
Supplement: Supplementary file 1 [file ijms-25-02459-s001.zip › ijms-2809436-SI.pdf]

Supplementary information

The Nuclear Localization Signal of Porcine Circovirus Type 4 Affects the Subcellular Localization of the Virus Capsid and the Production of Virus-like Particles

Jiawei Zheng [1,†], Nan Li [2,†], Xue Li [1], Yaqi Han [1], Xinru Lv [1], Huimin Zhang [1] and Linzhu Ren [1,3,*]

[1] College of Animal Sciences, Key Lab for Zoonoses Research, Ministry of Education, Jilin University, 5333 Xi'an Road, Changchun 130062, China; zhengjw21@mails.jlu.edu.cn (J.Z.)

[2] Changchun Veterinary Research Institute, Chinese Academy of Agricultural Sciences, 666 Liuying West Road, Changchun 130122, China; linan226@126.com

[3] College of Veterinary Medicine, Yunnan Agricultural University, Kunming 650201, China

* Correspondence: renlz@jlu.edu.cn

† These authors contributed equally to this work.

This file includes:

Supplemental Table S1

Figure S1 to S2

Supplementary Table S1. Primers used for site-directed mutagenesis techniques.

Primer Name	Primer Sequence
pEGFP-CapΔNLS-A	F：ccgctcgagatgccaatccggggactg
	R：cggggtacctccctgtttggggtagttaac
pEGFP-CapΔNLS-B	F：ccgctcgagatgccaatcagatctaggtacagcagac
	R：cggggtacctccctgtttggggtagttaac
pEGFP-CapΔNLS	F：ccgctcgagatgggaattttccatgcgcgcttc
	R：cggggtacctccctgtttggggtagttaac
pET-28a-PCV4CapΔNLS	F：ggaattttccatgcgc
	R：gattggcatatggctg
pET-28a-PCV4capΔNLS-A	F：cggggactgtggcc
	R：gattggcatatggctgcc
pET-28a-PCV4capΔNLS-B	F：aacggaattttccatgcgc
	R：ccacagtccccgcc
pET-28a-PCV4CapΔNLSA1	F：cgtaaccggcggaaccag
	R：gattggcatatggctgccg
pET-28a-PCV4CapΔNLSA2	F：cggggactgtggccccgg
	R：cctccgtctgctgtacctagatctgattgg
pET-28a-PCV4CapΔNLS-Aβ	F：cggggactgtggccccgg
	R：ccgccggttacgcctccg
pET-28a-PCV4CapΔNLS-Aα	F：agcagacggaggcgtaac
	R：gattggcatatggctgcc

Figure S1

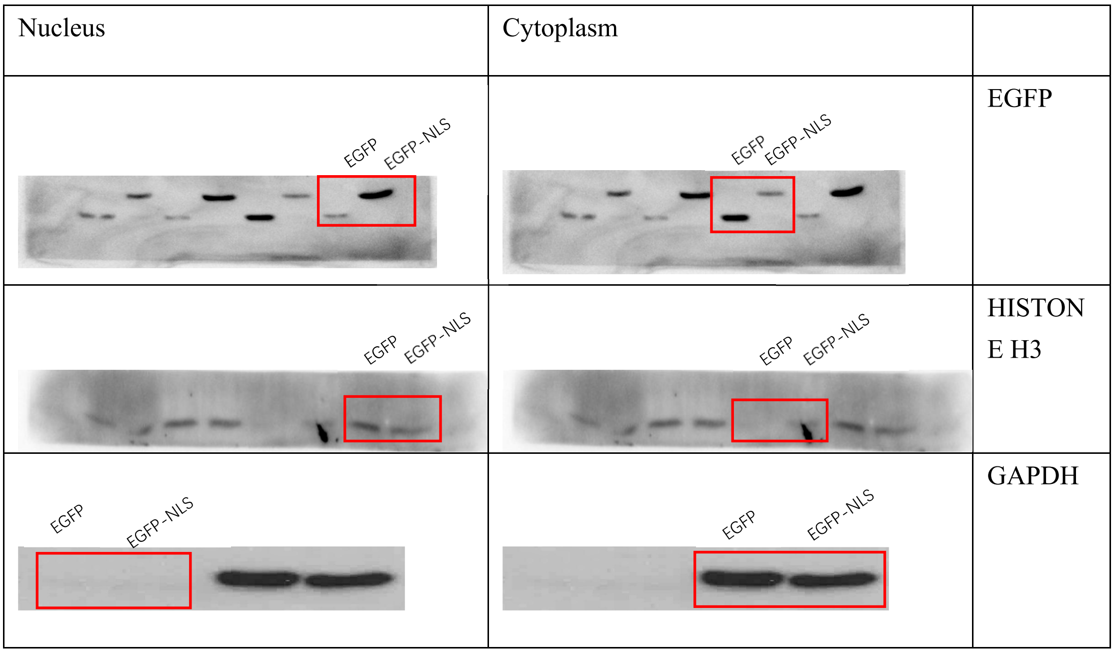

Figure S1. Original Western blot images used to prepare Figure 1C.

Figure S2

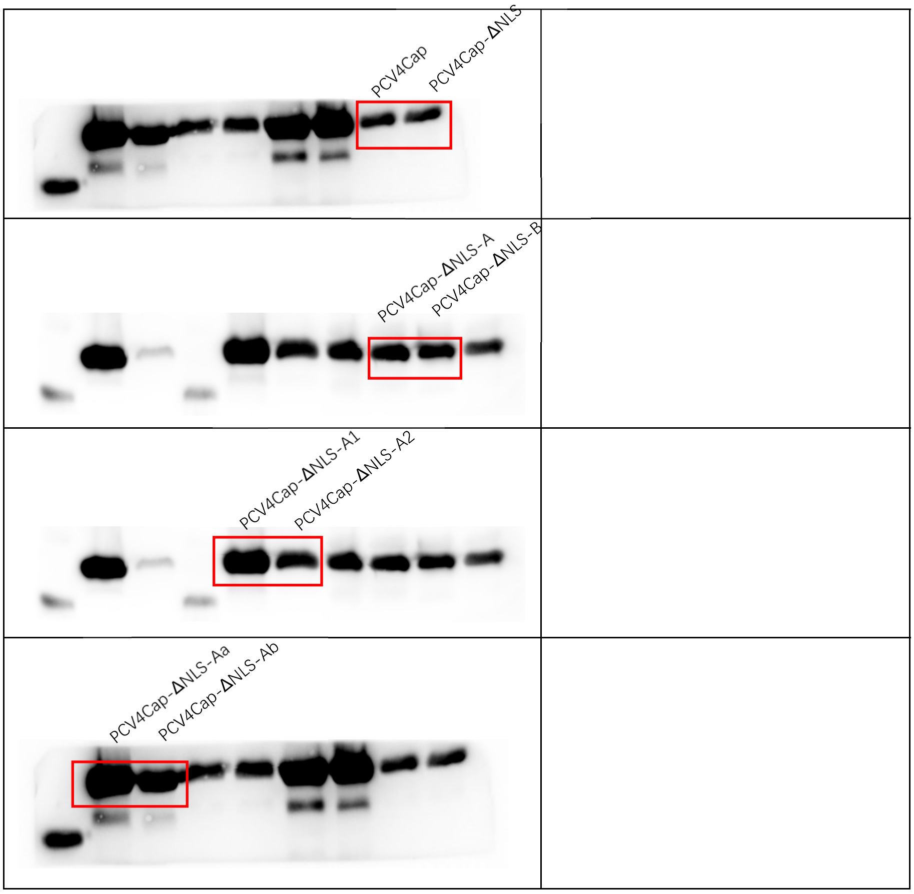

Figure S2. Original Western blot images used to prepare Figure 3B.
